# The influence of learning mode and learning sharing behavior on the synchronicity of attention of sharers and learners

**DOI:** 10.1186/s40359-022-00871-z

**Published:** 2022-07-04

**Authors:** Zhao Xiaojun, Kang Xinrui, Li Xupeng

**Affiliations:** School of Education, Hebei University, Baoding, People’s Republic of China

**Keywords:** Augmented reality learning, Academic emotions, Learning mode, Learning sharing behavior, Brain computer interface

## Abstract

Attention is the concentration of mental activities to a certain object, and students' inattentiveness in class directly affects their learning efficiency. As an emerging technology of educational application, augmented reality (AR) technology combines virtual reality and three-dimensional reconstruction to bring multisensory stimulation to students, enhancing immersion and attention in learning. A quantitative study was conducted on third-grade pupils. Study 1 examined whether learning mode and learning sharing behavior affect the synchronization of sharers’ and learners’ attention. Study 2 examined the impact of learning mode and sharing role on sharer and shared. The results showed that compared with learning alone, when sharing, the attention score of AR group is higher than that of text group. Whether it is the sharer or the shared, the attention score of AR group is higher than that of text group. AR has more advantages than text in terms of learning attention. In future research, it is optional to diversify AR learning materials and further use near-infrared spectroscopy technology to study interactive learning in AR mode.

## Introduction

### Students' efficient learning

With the continuous popularization of new education and teaching ideas, improving students' learning efficiency has been widely considered, which is the guarantee for students to maximize their energy and efforts [1]. The efficient completion of students' learning tasks needs to suppress the interference of irrelevant stimuli, and maintaining attention to the target task is very important for the individual's learning and development [2]. Childhood is the peak period of attention deficit problems and deserves high attention. Previous studies on attention-deficient children have confirmed a high correlation between learning attention and academic performance, and insufficient attention leads to poor academic performance [3]. Attention is an indicator of brain efficiency, affecting memory storage, retrieval and maintaining attention while learning can achieve effective knowledge acquisition [4].

As a nonintellectual factor affecting students' efficient learning, academic emotion is a variety of emotions generated by students engaged in learning-related activities. Such emotions may include students' emotional experiences during classroom learning, daily homework, or exams, and may be divided into positive academic emotions (pride, happiness, contentment, relaxation) and negative academic emotions (anxiety, anger, boredom, helplessness, depression) [5]. Due to the limitation of traditional classroom resources and space, the way for learners to obtain learning information is relatively simple, and boring learning content can easily lead to negative academic emotions and loss of interest in learning [6]. Reducing interest in learning easily shifts attention from current learning activities to other affairs, which is not conducive to learning activities [7].

Immersion theory (flow theory) was originally proposed by Mihaly Csikszentmihalyi in 1975 and refers to the whole-hearted devotion to an activity to achieve a state of full attention and high concentration [8]. Augmented reality (AR) is a technology that superimposes virtual things generated by computers into the real environment, which can realize the combination of virtual and real, and enhance people's perception of real items through intelligent means [9, 10]. Users interact with objects presented by AR devices, which easily achieves the full concentration of their mental state and can usually focus attention quickly and for a long time, which is an important feature of immersion experience [11]. Many studies have applied augmented reality to the field of education, especially for students in lower grades [12, 13]. Educational games have emerged, which use the combination of virtual and real to give students multisensory experience through visual three-dimensional imaging and sound to make the teaching content more realistic and improve the boring content [14]. Educational games reduce the difficulty of learning content, thereby enhancing students' attention to the current learning task and assisting students to complete the process of knowledge comprehension [15]. In addition, students can freely change the shape and position of the original model by touching, rotating, and interacting with it in real time to make students more immersed and enhance learning autonomy while improving learning interest [6]. It is difficult for the traditional classroom to maintain students' learning interest and continuity. The application of augmented reality technology in education provides a new possibility for the reform of the educational environment and has great practical significance.

Many studies have shown that human–computer interaction easily achieve immersion experience and enhances attention [11]. In addition, human interaction is also an essential link in learning. In learning tasks, the main form of human interaction is sharing behavior, which is a typical prosocial behavior that means that individuals are willing to share some resources with others, including specific and virtual resources [16]. The knowledge acquired by students in the learning process belongs to virtual resources, and the knowledge transfer among peers belongs to sharing behavior [17, 18]. In this study, knowledge sharing behavior refers to the behavior that students communicate in learning and transfer their learned knowledge to others. Students in lower grades can maximize their interest in learning content by interacting with their peers [19]. Both the sharer and the shared are in the process of knowledge sharing and can maintain a good learning state and benefit from interactive learning [20]. In particular, primary school students who have just come into contact with English have certain difficulties in learning English. If they carry out effective interactive learning, the state of learning English may be better.

There are many factors for individual knowledge sharing behavior [21]. The cognitive evaluation theory of emotion points out that the process of emotional evaluation will be affected by two factors: internal psychological structure and external environmental stimulation; its theoretical framework is "event-emotional experience-behavior" [22, 23]. When stimulating events lead to people's satisfaction and happiness, they have a higher willingness to share, which easily leads to altruistic sharing behavior; when feeling depressed and unhappy, it is not easy to lead to altruistic sharing behavior [24]. At this stage, game-based learning (GBL), as a supplement to traditional teaching, has been widely recognized by primary school students [25]. The immersive technology of three-dimensional, real-time interaction and multisensory experience provided by augmented reality is not common in the traditional classroom and is used to make up for its limitations. The application of new technology can stimulate students' learning interest and enthusiasm to a certain extent and promote a sense of pleasure in the learning process [6]. Emotion is the inherent physiological and psychological characteristic of human beings, and sharing behavior is affected by emotional potency. Under positive emotional experience, students have a higher willingness to share and are more likely to have sharing behavior.

### Learning activities based on brain-computer interfaces

With the development of artificial intelligence in neuroscience and other fields, brain-computer interface (BCI) technology has gradually developed. The brain is the control system of the human body, and the information sent out can be transmitted through Electroencephalogram (EEG). People will have corresponding changes in EEG signals under different stimuli and scenes [26]. In systems based on brain-computer interfaces, compared with artificial intelligence technology that relies on physiological signals such as skin electricity and heart rate to identify personal states, EEG directly connected with brain-computer interfaces can better reflect personal emotional states [27]. Therefore, EEG has become the connection between learners' brains and computers and is the key to observing people's brain function. Based on the human brain, the brain-computer interface can instantly reflect the brain activity status, such as attention, tension or relaxation. At the same time, the brain-computer interface can also actively adjust personal concentration to realize the control of brain function data to realize brain-computer interaction. Therefore, the brain-computer interface is also called the "brain control" system [28]. Modern brain-computer interface technology is divided into invasive and noninvasive technologies. Because of its safety and convenience, it is noninvasive and has been widely used in medical rehabilitation, real-time monitoring of athletes' status, military, daily home and other fields [29].

The combination of BCI technology and education is reflected in the development of intelligence games and classroom monitoring. For example, Lin and Kao [30] use brain computer interface technology to obtain students' learning state in class learning based on EEG signals and propose that the identification of learning psychological state is helpful to improve students' learning effect and teachers' teaching methods. At present, more research focuses on the attention of students' learning process, which is an important index to reflect students' cognitive level. The largest test of whether AR can occupy a place in education lies in the quality of classroom teaching in the AR environment. In the investigation of primary and middle school students' classroom teaching, it is found that students' attention is an intuitive reflection of whether the curriculum attracts students and can objectively reflect the quality of classroom teaching [31]. Bronack mentioned that augmented reality software and other immersive learning media, such as teaching games and virtual spaces, can give learners a sense of presence and enhance their intuition and attention [32]. Attention is the intensity of students' attention to a certain object, which is manifested in the inhibition of interfering stimuli and an effective indicator of students' learning state [33]. Some studies have shown that academic emotion is closely related to attention. Emotion affects students' allocation of attention resources and then affects students' processing process of related tasks [34]. Emotion can also affect the selective attention and sustained attention of adolescents with learning disabilities [35].

In this study, the visualization of physiological signals and attention in the learning environment, can more accurately reflect whether there are differences in the learning states held by students under two different teaching modes. A better learning state in the AR environment will promote the application of AR in education. Therefore, combined with the Brainlink Pro system developed by the Hong-zhi-li company, this study can accurately collect the brain function data of learners in learning activities including attention [36, 37]. The system is safe, flexible, easy to use, has high tolerance to head movement, can also well balance the characteristics of primary school students' hyperactivity, better fits the real learning environment of primary school students, and has higher ecological validity.

The study investigates whether the AR learning mode and learning sharing behavior affect the synchronicity of the attention of sharers and learners. The study makes the following hypotheses. (1) The learning mode affects learning attention, and the attention of the AR group is higher than that of the text group. (2) Learning sharing behavior affects learning attention, and the attention of the sharing group is higher than that of the learning group. (3) Sharing role (sharer and shared) does not affect learning attention.

## Study 1: Impact of different learning modes on learning or sharing attention

### Participants

Data were collected through cluster random sampling technique. G*Power is software for sample size calculations of various statistical methods [38]. In this study, we used G*Power 3.1 to calculate the sample size. We set the effect size to 0.3, α to 0.05, and the power of the test to 0.80 and obtained a calculated sample size of 24. A total of 80 third-grade students from a primary school in Hebei Province were randomly divided into an AR learning group (AR sharing group) and a text learning group (text sharing group). The participants were divided into two groups with equal opportunities. All the collected data with incomplete learning operations and unstable data connections were deleted. After excluding the invalid data, there were 58 valid data (25 girls), with an average age of 9.05 years (SD = 0.394 years), including 29 in each group (Table 1). All participants had normal naked or corrected visual acuity. In addition, this study obtained informed consent before the implementation of this study.

**Table 1:** Basic information

Learning model	Learning sharing behavior	Gender	Quantity
Augmented reality	Learning (sharing)	Male	16
		Female	13
Text	Learning (sharing)	Male	17
		Female	12
Total			58

### Experimental materials

**Fig. 1:**
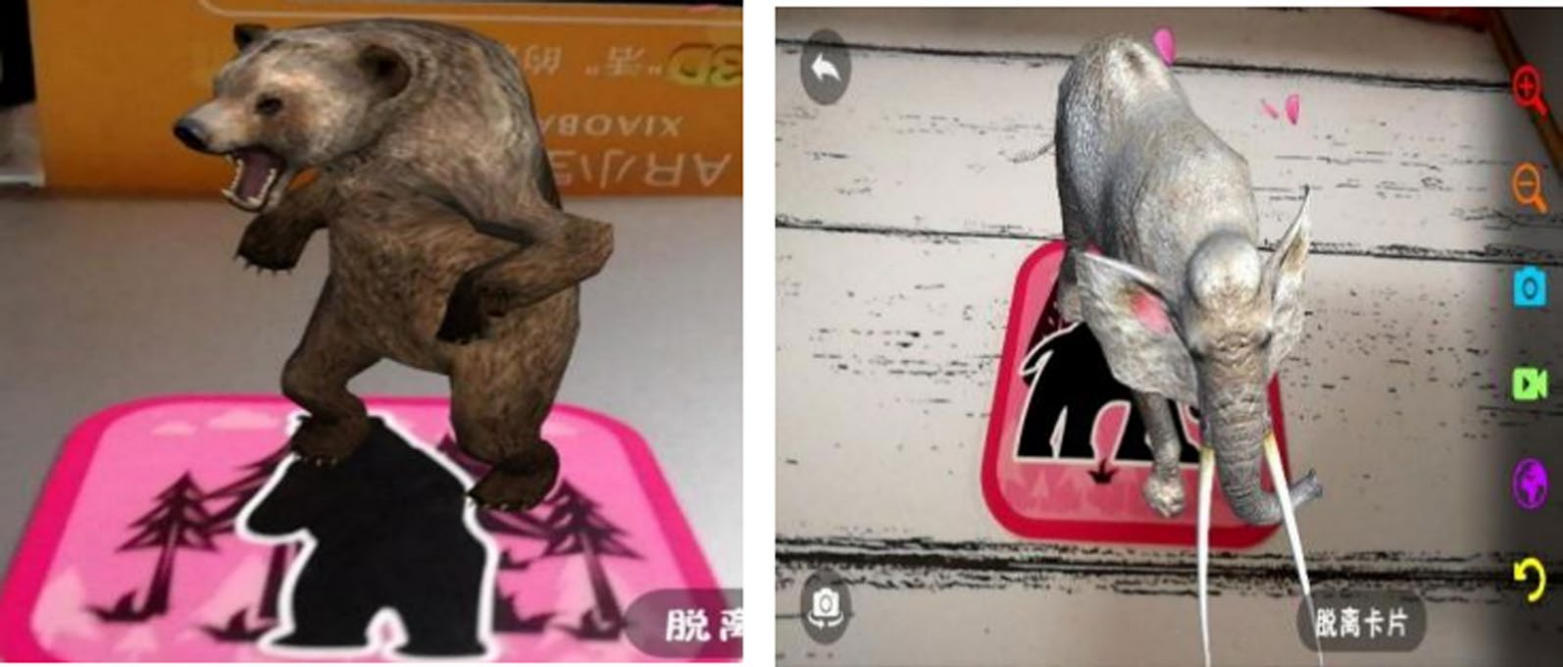
AR materials

AR materials (Fig. 1): AR cards and smartphones equipped with the Xiaobao Zoo app. The AR cards are an English learning product based on AR technology developed by Zhihe Qingyang Technology Co., Ltd. (China). There are 64 AR cards with Chinese and English words of animals on the front and corresponding animal pictures on the back. In this study, 12 animal cards were selected. The words in these cards come from the 12 animal words in the textbook for third graders this semester (Hebei Education Press). Students scan animal pictures through mobile phones for AR learning. After scanning, students can see three-dimensional animal images (visual), accompanied by animal calls (auditory), Chinese, English, Russian and Korean pronunciation and animal habits. At the same time, students can create interactions by interacting with the screen. On this basis, students can choose what they need to learn and become the subject of learning to a certain extent.

**Fig. 2:**
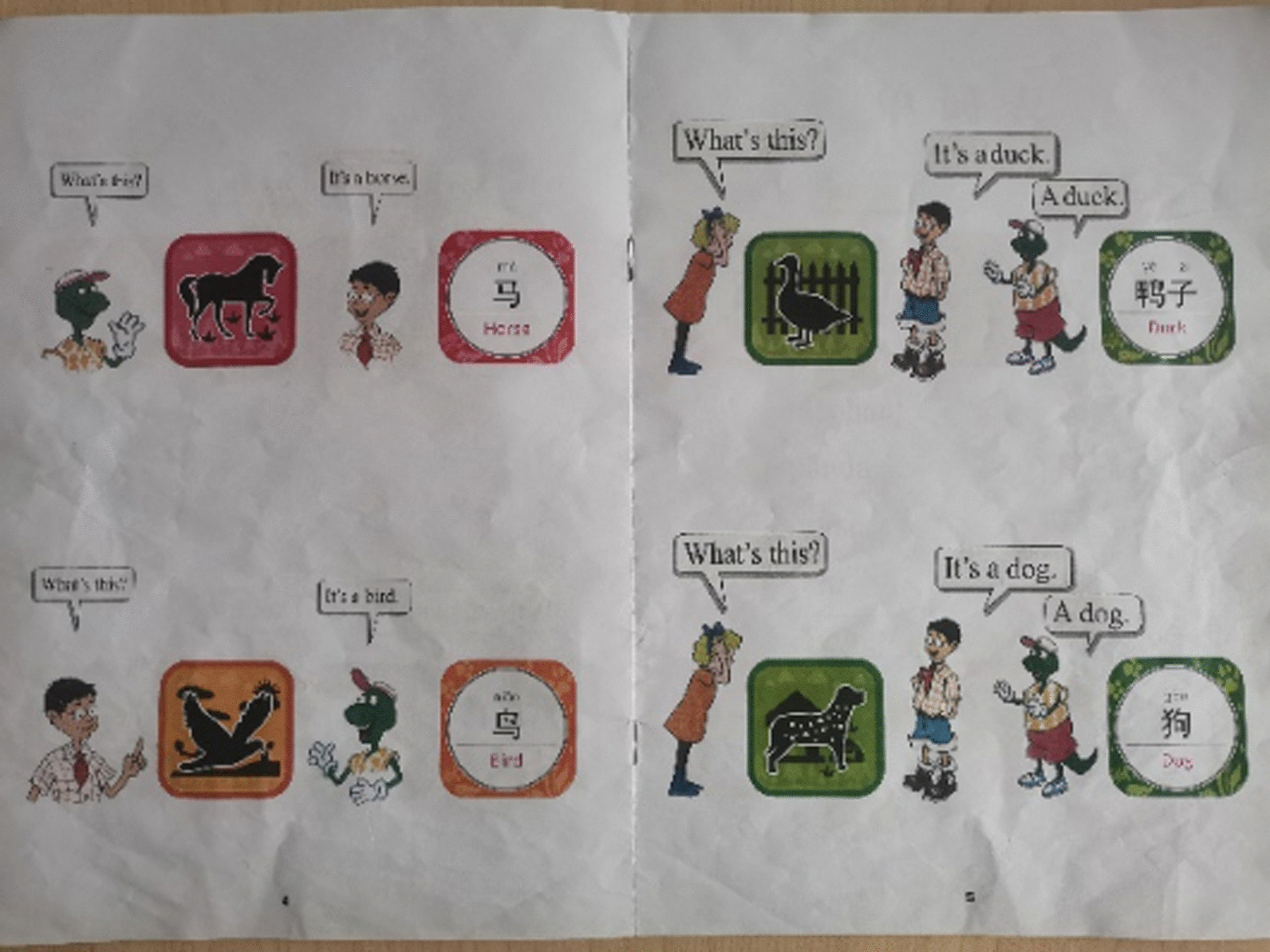
Text material

Text material (Fig. 2): an experimental book made in imitation of the English textbook of Hebei textbook edition. The book is printed with the same front and back patterns as 12 AR cards. To be closer to the learning environment of primary school students' English text, the book also adds the images of Danny, Jenny and Li Ming, as well as the simple dialogue between the three people based on words such as "this is an elephant".

**Fig. 3:**
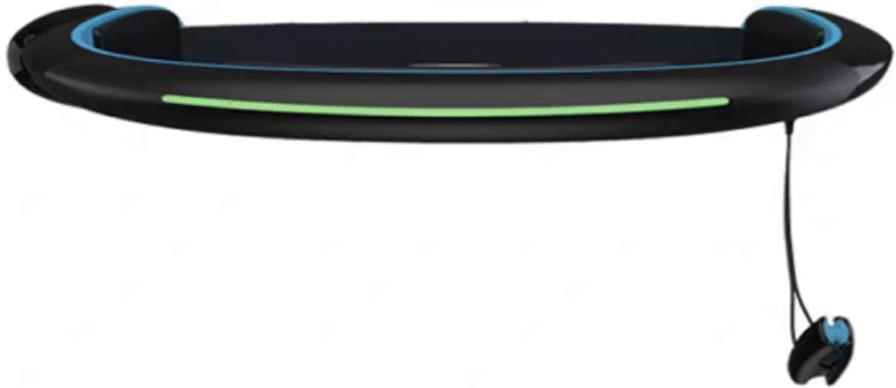
Brainlink Pro system

Brainlink Pro system based on brain-computer interface technology (Fig. 3): It is a wearable device that adopts the product developed by Hong-zhi-li Technology Co., Ltd. During the task, the device receives human EEG signals through the forehead of the brain at an output frequency of 1 Hz per second, which is filtered by the built-in sensor, and the readable parameters are obtained through chip analysis. Then, the signals are transmitted to the terminal device through the Bluetooth module. The experiment adopts attention parameters. The data range parameter is 1–100. The higher the data value is, the more focused the learner is. 0–20 indicates very low levels of attention, 20–40 below normal, 40–60 normal, 60–80 above normal, and 80–100 very high [39].

### Experimental design

The study conducted a 2 (learning mode: AR vs. text) × 2 (learning sharing behavior: learning vs. sharing) mixed experimental design, in which the learning mode was a between-subject variable and the learning sharing behavior was a within-subject variable. The dependent variable is attention level. The study conducted a quasi-experimental research design.

### Experimental procedure

Preparation stage: The experiment was conducted in a quiet classroom. There were no other people except the researchers and participants. The researcher has a CET-4 or CET-6. The AR sharing group researchers will explain the AR equipment and operation methods. After the explanation, 2–3 students were extracted to operate by themselves. After the operation, the researchers will explain the precautions during the experiment to the participants and start formal learning after ensuring that there is no doubt.

Learning stage: in the AR sharing group, participants needed to use AR equipment and cards to learn 12 English words for 8 min; in the text sharing group, participants used text materials to learn the same 12 English words for 8 min.

Sharing stage: after learning, let the two groups of participants who learned words by AR and text in the learning stage share formally with the students who did not participate in the learning for one minute. During the process, we collected the Brainlink Pro brain function data (attention value) of the sharer and shared. After the experiment, the device was removed from the subject's head.

### Data analysis

SPSS statistics 25.0 was used for data management and analysis. When processing the data, repeated measures analysis of variance was used to test the main effect and interaction of learning mode and learning sharing behavior on attention.

### Results

**Fig. 4:**
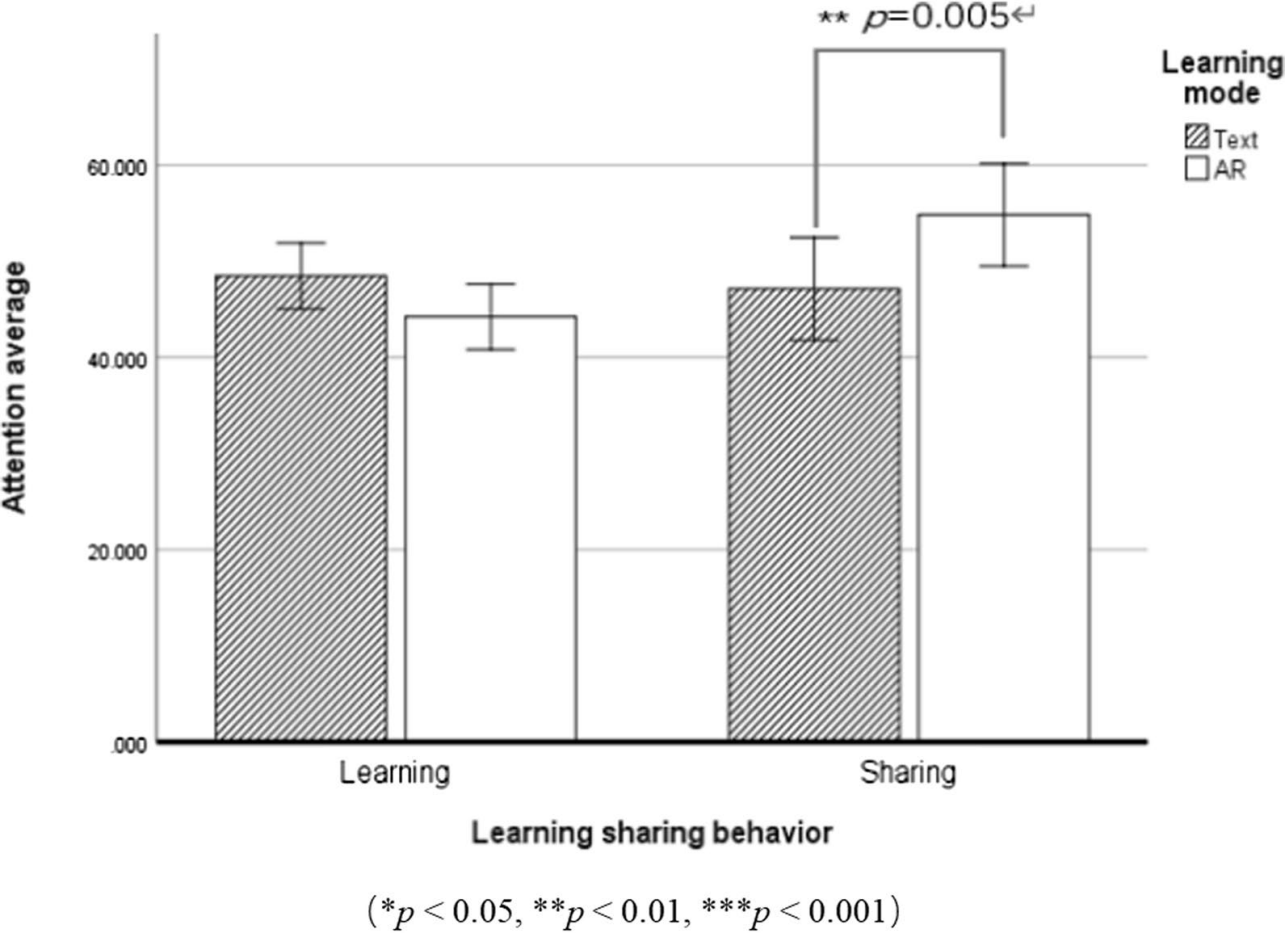
Attention under the learning mode and learning sharing behavior

The learning mode and learning sharing behavior were taken as independent variables, and attention level was taken as the dependent variable for repeated measures analysis of variance. The descriptive statistical results are shown in Table 2. The results showed that in the main effect of learning sharing behavior was low significant in terms of attention,* F* (1,56) = 4.998, *p* = 0.029, *η*_*p*_^2^ = 0.082, specifically as follows: the attention score of the sharing group (*M*_sharing_ = 50.940, *SD*_sharing_ = 14.744) was significantly higher than that of the learning group *(M*_learning_ = 46.182, *SD*_learning_ = 15.830). The main effect of learning mode was low significant, *F* (1,56) = 0.523, *p* = 0.472, *η*_*p*_^2^ = 0.009. The interaction between the two was significant, *F* (1,56) = 8.344, *p* = 0.005, *η*_*p*_^2^ = 0.130. The simple effect analysis (Fig. 4) showed that when sharing, the attention score of the AR group (*M* = 54.788, *SD* = 2.665) was higher than that of the text group (*M* = 47.091, *SD* = 2.665).

**Table 2:** Descriptive statistical analysis of attention during sharing

Learning mode	Learning sharing behavior	Attention	
		*M*	*SD*
Augmented reality	Learning	44.214	1.708
	Sharing	54.788	2.665
Text	Learning	48.439	1.708
	Sharing	47.091	2.665

## Study 2: Impact of different learning modes on sharer or shared attention

### Participants

Data were collected through cluster random sampling technique. In this study, we used G*Power 3.1 to calculate the sample size [38]. We set the effect size to 0.3, α to 0.05, and the power of the test to 0.80 and obtained a calculated sample size of 90. A total of 160 third-grade students from a primary school in Hebei Province were randomly divided into an AR sharer group, text sharer group, AR shared group and text shared group. The participants were divided into four groups with equal opportunities. After excluding the invalid data, there were 116 valid data (53 girls), with an average age of 9.10 years (SD = 0.425 years), including 29 in each group (Table 3). All participants had normal naked or corrected visual acuity, and informed consent was obtained before the implementation of this study.

**Table 3:** Basic information

Learning model	Sharing role	Gender	Quantity
Augmented reality	Sharer	Male	16
		Female	13
	Shared	Male	15
		Female	14
Text	Sharer	Male	17
		Female	12
	Shared	Male	15
		Female	14
Total			116

### Experimental materials

All experimental materials are the same as in study 1.

### Experimental design

The study conducted a 2 (learning mode: AR vs. text) × 2 (sharing role: sharer vs. shared) between-subject experimental design. The dependent variable is attention level. The study conducted a quasi-experimental research design.

### Experimental procedure

After the two groups of participants in study 1 finished learning, they used AR and text to share with those who did not participate in the learning. During the process, we collected the Brainlink Pro brain function data (attention value) of the sharer and shared.

### Data analysis

SPSS statistics 25.0 was used for data management and analysis. When processing the data, two-factor analysis of variance was used to test the main effect and interaction of learning mode and sharing role.

### Results

The descriptive statistical results are shown in Table 4 in the sharing process. The learning mode and sharing role were taken as independent variables, and attention level was taken as the dependent variable for analysis of variance. The study conducted a 2 × 2 between-subject analysis of variance. The results showed that in the process of sharing, the main effect of learning mode was low significant in terms of attention,* F* (1,112) = 6.002, *p* = 0.016, *η*_*p*_^2^ = 0.051, specifically as follows: the attention score of the AR learning group when sharing (*M*_AR_ = 53.128, *SD*_AR_ = 14.528) was significantly higher than that of the text group *(M*_text_ = 46.182, *SD*_text_ = 15.830); the main effect of sharing role was low significant, *F* (1,112) = 0. 821, *p* = 0.367, *η*_*p*_^2^ = 0.07, and the interaction between the two was low significant, *F *(1,112) = 0.070, *p* = 0.792, *η*_*p*_^2^ = 0.01.

**Table 4:** Descriptive statistical analysis of attention during sharing

Learning mode	Sharing behavior	Attention	
		*M*	*SD*
Augmented reality	Sharer	54.788	14.171
	Shared	51.468	14.939
Text	Sharer	47.091	14.528
	Shared	45.273	17.245

## Discussion

### Impact of different learning modes on learning or sharing attention

The interaction between learning mode and learning sharing behavior was significant, and there was low significant difference in attention scores between the AR group and the text group when individuals were learning alone. When sharing, the AR group had a higher attention score than the text group. Statistically significant and with a moderate effect size indicate that the attention of the AR group and the text group is significantly different when sharing [40]. Hypothesis 1 and hypothesis 2 were supported. Compared with the learning process, students in the sharing process engage in more interactive learning with their peers, and the interaction with learning is more social, which can meet the needs of students' mutual cooperation and greatly promote students' attention. The results are in line with the child psychologist Jean Piaget's view that collaborative learning is a main way to promote the construction of children's cognitive development. Students promote each other's learning in the form of groups or teams, which can mobilize students' learning enthusiasm to a great extent [41], which is consistent with the result that a computer-supported collaborative writing environment can improve students' concentration [42]. In some studies on children in special groups, it has been found that AR learning mode will result in higher positive academic emotion and attention. Therefore, the emergence of this contradictory result indicates that the effect of AR learning mode may be related to the selected groups of subjects and the experimental materials [43, 44]. Therefore, in order to better understand the impact of AR learning mode on different primary school students, we can try to use different materials in different groups of primary school students in future research. Under collaborative learning supported by augmented reality, students' attention is higher. Compared with VR technology, AR technology can better support collaborative learning. Students can experience and interact with words and sounds superimposed on real things through AR technology at the same time [6]. The collaborative learning environment built by AR technology for students conforms to students' interests. Students are willing to share this learning method with others. When sharing, they can focus more on the current task and are less disturbed by the outside world. In the process, the immersive experience provided by AR makes collaborative learning more beneficial, which is no longer limited to the interactive cooperation of traditional paper books and can effectively activate students' attention level.

### Impact of different learning modes on sharer or shared attention

The main effect of learning mode on the attention of sharer and shared was low significant, with a moderate effect size, indicating a large difference between the two groups [40]. In the process of sharing, the attention of students in the AR group was significantly higher than that in the text group; that is, whether sharing or being shared, the attention of students in the AR environment was significantly higher than that in the text environment. Hypothesis 1 and hypothesis 3 were supported. Sharing behavior is the embodiment of positive academic emotion, indicating that an AR learning environment can activate more positive academic emotions, which to some extent shows that the AR learning method is novel, can arouse students' interest in learning, and is widely loved by primary school students [45]. The sharing role did not affect attention level. Regardless of sharing or shared, the degree of attention is consistent. Sharing behavior is a typical prosocial behavior, which means that individuals are willing to share some resources with others [16]. In this study, sharing or being shared is essentially an invisible interpersonal interaction. Due to the influence of interaction, the attention focus and attention level of both parties are maintained at a relatively good level. In particular, peers, as helpers of mutual learning, can effectively feel that they have become the subject of learning in the process of discussion and sharing so that students can improve the level of concentration in the process of sharing and being shared. However, it is not excluded that there are other possibilities under the same results, such as based on Plickers software, each student can participate in the interactive learning with the teacher, which can significantly improve the students' learning engagement, and it is possible to join teachers' interactive learning to improve students' attention [46]. A meta-analysis shows that only by providing students with more opportunities for cooperation, exchange and participatory learning can AR technology be brought into full play [47]. Future research can expand the sample size and use more rigorous experiments to determine the generalizability of the results.

### The positive role of augmented reality learning in learning activities

An intelligent learning environment is a learning place or space with the characteristics of evaluation, learning resources, learning situations, interaction and recording learning processes [48] and is an inevitable trend of learning environment reform. The support of new technologies can provide strong support for the construction of intelligent learning environments. The effect of augmented reality on the intelligent learning environment is that it breaks the restrictions of time and place in the traditional classroom and has obvious educational advantages, including three-dimensional presentation, knowledge interaction, immersion experience and real-time feedback, so that AR teaching can provide a highly effective intelligent learning environment [45]. However, it is conditional for pupils to benefit from the AR environment. Individuals did not show an advantage in attention when using AR alone, but it is undeniable that AR can promote the attention of peers when sharing learning. From the perspective of pupils' physical and mental development, pupils' learning status is highly situational, and different specific situations in the classroom may cause students to have various emotional experiences, which will affect learning [49]. AR learning has the characteristics of high immersion and interaction so that learners can devote themselves to it, feel a higher sense of participation and control in interactive learning with peers, which plays a certain role in cultivating pupils' positive academic emotions [50]. This makes it possible for AR to be applied to education.

### Deficiencies and prospects

Our study has several limitations. Firstly, the experimental materials of this study are relatively preliminary, and future research needs to develop diversified AR materials suitable for this age group to explore whether the benefits of AR in sharing learning are universal among different disciplines and grades. Previous studies have found that the impact of augmented reality learning on the learning effect of primary school students is relatively poor in each learning stage [47]. Another study came to the opposite conclusion [51]. We believe that this result may be related to the design of learning materials. Whether only developing AR learning materials that are more suitable for pupils' cognitive ability and use habits will bring better learning effects to pupils deserves further research. Second, although this study found the advantages of AR in peer sharing learning, it did not reflect the one-to-one correspondence between the sharer and the shared. Further research can be studied in more detail by using near-infrared spectroscopy to realize the sharer and shared of two-person interactive information synchronous collection.

## Conclusion

(1) Collaborative learning supported by AR has positive significance for maintaining pupils’ attention. (2) In the process of collaborative learning, both the sharer and the shared can stay attention in the AR environment.

## Data Availability

The datasets generated and/or analysed during the current study are not publicly available due subjects are under 16 but are available from the corresponding author on reasonable request.
